# Task-shift Model in Pre-hospital Care and Standardized Nationwide Data Collection in Japan: Improved Outcomes for Out-of-hospital Cardiac Arrest Patients

**DOI:** 10.31662/jmaj.2020-0074

**Published:** 2021-01-14

**Authors:** Shinji Nakahara, Tsuyoshi Nagao, Ryuichi Nishi, Tetsuya Sakamoto

**Affiliations:** 1 Graduate School of Health Innovation, Kanagawa University of Human Services, Kawasaki, Japan; 2 Department of Emergency Medicine, Teikyo University School of Medicine, Tokyo, Japan

**Keywords:** task shift, paramedics, first-aid, pre-hospital care, out-of-hospital cardiac arrest

## Abstract

Out-of-hospital cardiac arrest (OHCA) is a growing worldwide public health concern. Previously, Japan experienced poorer outcomes among OHCA patients than in other high-income countries. In the early 1990s, through policy changes, the Japanese government introduced a task-shift model in pre-hospital care. Some medical practices previously provided by physicians exclusively were delegated to non-physicians, including laypeople. Additionally, we initiated a nationwide data collection system for evaluation. We started a nationwide registry of OHCA patients, a paramedic system to provide advanced life-support care, and basic life-support training for laypeople. In the 2000s, the procedures paramedics could provide were expanded, laypeople were allowed to use automated external defibrillators, and the Utstein style was introduced to the national registry.

Consequently, pre-hospital advanced care and bystander first-aid increased, registry-based research contributed to evidence-based practices, and―most importantly―outcomes of OHCA patients considerably improved. These Japanese experiences demonstrate that streamlining pre-hospital care, including bystander interventions and standardized data collection, can improve OHCA patient outcomes. Despite this progress, however, there still exist many issues to be addressed in response to the changing and increasing care demands within Japan’s aging population.

## 1. Background

Out-of-hospital cardiac arrest (OHCA) is a growing global public health concern ^(1), (2)^. Prompt response by bystanders and emergency medical services (EMS) can save a considerable number of OHCA patients; however, such prompt response requires increased awareness and training among laypeople, as well as streamlining the EMS system ^(3)^.

Japanese OHCA patients experienced worse outcomes than those in other high-income countries ^(4), (5)^. To tackle this issue, the Japanese government began various measures in the early 1990s based on policy change. The government introduced a task-shift model in pre-hospital care, including advanced life-support procedures, performed by paramedic-level EMS personnel, and defibrillation performed by laypeople using an automated external defibrillator (AED) ^(6), (7)^. Previously, these procedures were provided exclusively by physicians. Concurrently, the government started nationwide data collection on OHCA patients for evaluation and research.

Consequently, patient outcomes improved, and evidence from Japan significantly contributed to developing and updating international guidelines. In this paper, we describe policy changes in Japan, which could benefit other countries with low OHCA survival rates due to insufficient pre-hospital care.

## 2. Organizational Context

The emergency care and EMS systems in Japan were organized in the 1960s and 1970s, based on legal and policy frameworks, in response to a rapid increase in road traffic injuries ^(8)^. The Fire Services Act, amended in 1963, mandated municipal fire departments to provide EMS (previously, fire departments provided EMS without a clear legal stipulation). The Fire and Disaster Management Agency (FDMA) became the national lead agency for EMS and determines policy, guidelines, and training curriculum for pre-hospital care. The policies achieved nationwide EMS coverage as an emergency transport system. Each municipal EMS system has a command center, and the phone number 1-1-9 is used for EMS calls nationwide. Generally, an EMS unit comprises three basic-level emergency medical technicians (EMTs-basic), who have completed 135 hours of training.

The Ministry of Health and Welfare (MHW) issued a ministerial ordinance in 1964 ^(9)^ to designate emergency care hospitals where EMS could transport patients. In 1977, MHW ^(10)^ created a masterplan to reorganize emergency care hospitals into three levels―primary, secondary, and tertiary―based on their ability to streamline referral networks and enhance hospital capabilities through subsidies. Tertiary emergency care was provided in Emergency Critical Care Centres, accredited by MHW.

## 3. Problem

Despite vigorous efforts to develop an EMS system and improve hospital care quality, pre-hospital care―including bystander first-aid―was quite primitive in the 1980s. One reason was EMTs-basic could perform only basic life-support procedures, such as bag–valve–mask ventilation and chest compression. At the time, the Medical Practitioners Act did not allow non-physicians to perform medical procedures, including diagnoses and treatments. Even auscultation with a stethoscope was not allowed. Additionally, laypeople were not well-trained in first-aid procedures. Bystanders could do nothing while waiting for an ambulance, and EMTs-basic could do little in the ambulance.

In the 1980s and early 1990s, Japan’s resuscitation science was still in its infancy. There was no standardized data collection mechanism to provide a basis for evaluation and evidence-based practices. Technical terms were not clearly defined, and various inclusion criteria were used without clear definitions in Japanese OHCA studies. Most physicians and researchers referred to OHCA patients as “dead-on-arrival” (DOA) or cardiopulmonary arrest on arrival (CPAOA) ^(5)^. These terms imply that those who achieved the return of spontaneous circulation (ROSC) before hospital arrival were not focused on by physicians or researchers ^(11)^. In fact, most reports on OHCA patients during this period, were hospital-based and focused on DOA/CPAOA; ^(4), (5)^ few studies reported population-based data on OHCA patients ^(12)^. Contrastingly, contemporaneous studies in other countries did focus on OHCA patients ^(5)^.

During this period, studies in Japan reported extremely poor prognoses among DOA/CPAOA, though various endpoints were used without clear definitions due to the lack of standardized data format. Full recoveries, a commonly used endpoint defined as returning to normal social activities, were usually around 1% of treated patients ^(4), (5)^. Patients who achieve ROSC before hospital arrival usually have better prognoses; however, they were excluded from DOA/CPAOA studies. However, even taking this bias into account, reported prognoses were poor compared to other countries ^(5)^.

## 4. Solutions

### 4.1. Pre-hospital care

Poor outcomes for OHCA patients, combined with a growing number of OHCA patients due to the rapid aging of Japan’s population, increased the need for improved pre-hospital care. An aggressive media campaign, started in 1989 to arouse public opinions, called for a task-shift model in pre-hospital care. This model would delegate some medical practices, such as defibrillation, to EMS personnel (Table 1) ^(13), (14)^. The campaign illustrated the effectiveness of the task-shift model for paramedics in the United States and compared it to the Japanese system ^(14)^. However, this required new laws, as the Medical Practitioners Act strictly prohibited task shifting for medical care.

**Table 1.** Policy Changes In the 1990s and 2000s.

Policy process	Policy content			
	Task shift in pre-hospital care			Evaluation/research
	EMT*	ELSTs	Laypeople	
1990s				
Media campaign started in 1989 MHW established the Commission for Emergency Care System in in 1989 The commission issued a report in 1990 FDMA established a working group for pre-hospital care in 1990 The group issued a report in 1990 Lawmakers decided to introduce a task-shift model, in which EMS personnel can provide some advanced procedures, and to enhance laypeople training	Guidelines for pre-hospital care procedures amended in 1991 EMTs-intermediate were allowed to: Use an automatic CPR machine Continue home-based treatments in the ambulance Use pneumatic-anti-shock garments Auscultate Monitor blood pressure, SpO_2_, and ECG Insert nasal airways Remove foreign bodies using laryngoscope and Magill forceps	ELST Act enacted in 1991 and ELST system started in 1992 Under on-line physician instructions, ELSTs were allowed to: Place intravenous lines Insert supraglottic airways Perform defibrillation using a semi-AED	FDMA Guidelines on training curriculum for laypeople and instructors issued in 1993 (training of BLS procedures)	Standardized data collection activities started Population-based studies using the Utstein style initiated (early 1990s) The FDMA national registry started in July 1994 The Utstein Osaka Project started in 1998
2000s					
Joint Commission for ELSTs’ services established in 2002 (by the health ministry and FDMA) The Commission delivered a recommendation to expand ELSTs’ roles Commission for Sophistication of Pre-hospital Care recommended introduction of the Utstein style to the national registry		Ordinance for Enforcement of the ELST Act amended Defibrillation with an AED without on-line instructions started in 2003 Endotracheal intubation under on-line instructions started in 2004 Adrenaline administration under on-line instructions started in 2006	The health ministry issued a notice in 2004 stating laypeople can use an AED in emergency situations, if a physician is not availabl Deployment of public-access AED started and increased	International Guidelines introduced in 2000 Establishment of Japan Resuscitation Council in 2002 SOS-KANTO study started in 2002 Establishment of Resuscitation Council of Asia in 2005 The Utstein style was introduced into the national registry in 2005 Resuscitation Council of Asia became a member of ILCOR in 2006

AED, automated external defibrillator; CPR, cardiopulmonary resuscitation; ECG, electrocardiogram; ELST, emergency life-saving technician; EMS, emergency medical service; EMT, emergency medical technician; FDMA, fire and disaster management agency; ILCOR, International Liaison Committee on Resuscitation; MHW, Ministry of Health and Welfare; SOS-KANTO, Survey of Survivors after Out-of-Hospital Cardiac Arrest in the Kanto Region; SpO_2_, peripheral capillary oxygen saturation *An EMT-basic, who has completed 135 hours of training, can perform basic life support procedures, suction of oral cavities, insertion of oral airways, and oxygen administration. An EMT-intermediate, who has completed 250 hours of training, can also do these procedures. ELSTs can do the procedures that EMTs can do. An EMT-basic can become an EMT-intermediate after additional 115 hours of training. One can become an EMT-intermediate after 250 hours of training without experience as an EMT-basic.

To address pre-hospital care improvement, MHW established the Commission for Emergency Medical Care in 1989. FDMA established a workshop group for pre-hospital care in 1990 (Table 1) ^(8), (13), (15), (16), (17)^. Both groups, after comparing pre-hospital care procedures and patient prognoses in Japan with those in other high-income countries, independently delivered the following three recommendations independently in 1990: 1) introducing and strengthening a physician-staffed ambulance system so that physicians could start advanced care at the scene; 2) expanding pre-hospital care procedures provided by EMS (including introducing paramedics to provide advanced pre-hospital procedures under specific physician instructions via radio communication); 3) first-aid training for the general public.

However, MHW and FDMA had somewhat different views regarding the first two recommendations. MHW emphasized physician-staffed ambulances’ potential, reflecting cautiousness toward the task-shift model expressed by both the Japan Medical Association and the Japanese Society of Anesthesiologists. They had concerns regarding the quality of advanced procedures provided by non-physician EMS personnel with limited training (135 hours of training is far less than other medical professions) ^(14)^. MHW also proposed a new qualification scheme, so that sufficiently trained personnel could provide advanced care (i.e., paramedics). Contrastingly, though recognizing the potential of a physician-staffed ambulance system, the FDMA emphasized the feasibility of the task-shift model, given the shortage of emergency physicians, particularly in rural areas, and the benefits of modifying the existing nationwide EMS system. FDMA proposed expansion of the list of procedures EMS personnel could perform, reflecting positive opinions from the Japanese Association for Acute Medicine and Tokyo Fire Department, the largest municipal fire department.

Based on these debates, lawmakers decided to establish the Japanese paramedic system and expand the procedures EMS personnel could perform, reflecting the views of both groups and public opinion and enhancing the physician-staffed ambulance system. Physician-staffed ambulances have been introduced in limited areas, with little impact on the whole country. The Emergency Life-Saving Technicians (ELST) Act was enacted in August 1991, to introduce Japanese paramedics, called ELSTs ^(17), (18)^. Also, FDMA amended its pre-hospital care guidelines and procedures to introduce EMTs-intermediate, who complete 250 hours of training and can perform more procedures than EMTs-basic (Table 1).

At this time, ELSTs can perform a limited set of advanced life-support procedures on OHCA patients, including defibrillation using a semiautomatic defibrillator, under specific physician instructions via radio communication (Table 1) ^(6)^. Although defibrillation by ELSTs shortens the time to defibrillation, leading to better recovery ^(19), (20)^, the benefits of pre-hospital defibrillation were fewer in Japan than other countries in the 1990s, as physician instructions were required ^(21), (22)^. Cardiac rhythm conversion―from shockable to non-shockable―sometimes occurred while ELSTs awaited instructions ^(22)^.

Later, in the 2000s, MHW and FDMA established the Joint Commission for ELST services to review the performance of ELSTs and discuss enhancement of their roles (Table 1). The commission recommended expansion of the procedures ELSTs could perform, based on an evaluation of ELSTs’ performance: one-month survival of more than 1,000 OHCA patients could be attributable to ELSTs’ care ^(23)^. Then, MHW amended the Ordinance for Enforcement of the ELST Act so that ELSTs could perform more advanced procedures.

To educate the public on first-aid and cardiopulmonary resuscitation (CPR) skills, FDMA issued training curriculum guidelines in 1993 for laypeople and instructors ^(24)^. This action did not require legal amendments because anyone can perform basic life-support procedures. Municipal fire departments have taken charge of training laypeople.

Another important policy change in the 2000s was AED use by laypeople. Although AED use had been considered a medical practice exclusively provided by physicians, MHW changed its interpretation of the Medical Practitioners Act and stated that one-time AED use in an emergency is not a medical practice if no physician is available. Therefore, it can be performed by laypeople. Thus, layperson use of AED has been permitted since July 2004 ^(25)^.

### 4.2. Standardized data collection and analysis

The Utstein style, an international data collection standard, was introduced to Japan in the early 1990s. It has facilitated considerable research and policy evaluation on OHCA patients, making international, regional, and before-after comparisons easier. Furthermore, it has expanded researchers’ and clinicians’ viewpoints on treatment for DOA/CPAOA patients in the emergency room to the occurrence, prevention, and pre-hospital care of OHCA patients in communities; thus, the term DOA/CPAOA has become obsolete.

FDMA started national registration of all OHCA patients in July 1994 to evaluate emergency care improvements, particularly ELSTs’ performance. The registry has played a pivotal role in describing the nationwide occurrence and prognoses of OHCA patients. However, the registry database did not follow the Utstein style and lacked information on initial cardiac rhythms, etiology, and neurological functions required for standardized international comparisons. Therefore, the Commission for Sophistication of Pre-hospital Care in FDMA recommended the introduction of Utstein style for the national registry in 2003, and FDMA began using the Utstein style in the national registry in 2005 ^(26), (27)^.

Introduction of the Guidelines 2000, the first guidelines developed through international collaboration, led Japanese resuscitation experts to recognize the necessity of international cooperation in developing evidence-based guidelines. Subsequently, the Japanese Resuscitation Council (JRC) was established in 2002 as an organization to coordinate domestic activities―such as publication and training―and participate in international activities ^(28)^. The Resuscitation Council of Asia was established in 2005, representing Asia as a member of the International Liaison Committee on Resuscitation (an international collaboration on resuscitation science).

### 4.3. Results of policy changes

The Japanese paramedic system, ELSTs, started in April 1992 (Table 1) ^(6), (18)^. Candidates must pass the national MHW board examination to be certified as an ELST. To qualify for the board examination, a candidate should either be an EMT-intermediate with at least five years’ experience or 2,000 ambulance service hours and has completed six months of training or has graduated as an ELST candidate from a vocational school or university. ELSTs and EMS units with ELSTs have been increasing (Table 2). In 2018, 26,581 ELSTs were engaged in EMS; out of 5,179 EMS units, 5,132 units (99.1%) had at least one ELST in the three crews ^(26)^.

**Table 2.** Increasing Trends in Emergency Life-saving Technicians, Trained Laypeople, and Public-access AEDs

	EMS*			Trained laypeople*			Public-access AEDs†
Year	ELSTs in EMS units, n	EMS units (total), n	EMS units with ELSTs, %	Trained in 3-hour course, n	Trained in 8-hour course, n	Short course, n	Number of units sold, n
1992	483	4237	4.0			
1993	541	4229	5.2			
1994	1,369	4331	11.5	246,356	10,680		
1995	2,232	4387	16.6	395,045	19,212		
1996	3,338	4416	23.9	491,300	25,758		
1997	4,556	4483	29.7	589,798	33,670		
1998	5,846	4515	37.2	655,700	34,807		
1999	6,757	4553	44.8	797,979	41,135		
2000	8,016	4582	51.2	861,699	48,393		
2001	9,461	4,563	56.8	901,039	53,795		
2002	10,823	4,596	62.8	970,898	58,410		
2003	12,152	4,649	67.6	1,081,946	61,746		
2004	13,505	4,711	73.0	1,053,715	65,895		1,307
2005	15,317	4,751	78.3	1,147,904	68,081		10,961
2006	16,468	4,779	82.4	1,388,212	78,922		45,417
2007	17,218	4,846	86.3	1,499,485	72,843		96,545
2008	18,336	4,871	88.5	1,541,459	77,660		164,343
2009	19,368	4,892	91.0	1,490,246	75,926		218,050
2010	20,383	4,910	93.1	1,408,864	76,999		264,165
2011	21,268	4,927	94.3	1,345,591	79,959		310,075
2012	22,118	4,965	95.9	1,410,981	84,898	224,230	364,959
2013	22,870	5,004	96.8	1,392,325	50,547	325,476	428,821
2014	23,560	5,028	97.4	1,376,149	84,864	392542	516,135
2015	24,223	5,069	97.8	1,355,791	84,307	409347	602,382
2016	24,973	5,090	98.4	1,315,946	82,385	443,943	688,329
2017	25,872	5,140	98.9	1,287,848	88,659	558,454	784,467
2018	26,581	5,179	99.1	1,245,971	91,014	656,226	881,467

AED, automated external defibrillator; ELST, emergency life-saving technician; EMS, emergency medical service *Data source: Fire and Disaster Management Agency. [Report on emergency medical service and rescue, 2018]. Tokyo: the Agency; 2019. Japanese ^(26)^. † Data source: Tanabe S, Yokota H. [Promotion of AED use by citizens and handling of related information, Report of Fiscal Year. 2018]. Tokyo: 2019. [AED sales and deployments]. Sakamoto T, editor; p. 15-22. Japanese ^(29)^.

At first, procedures that ELSTs could perform were limited. They could place intravenous lines using lactate linger solution, insert supraglottic airways, and defibrillate using semiautomatic defibrillators on OHCA patients, all under specific physician instructions via radio (Table 1). They were not allowed to defibrillate without specific instructions, perform endotracheal intubation, or administer adrenaline. After that, the number of procedures ELSTs can perform eventually increased (Table 1). Defibrillation with an AED by ELSTs without specific physician instructions started in 2003. Endotracheal intubation and adrenaline administration by certified ELSTs began in 2004 and 2006, respectively. By 2018, 25,222 ELSTs had been certified for adrenaline administration and 14,609 for endotracheal intubation (including those not engaged in EMS).

Laypeople training provided by municipal fire departments started in 1994, based on FDMA guidelines. There are two training levels for laypeople. The three-hour basic course includes basic airway management, CPR, and removing a foreign body from the airway. In addition to these basic skills, the eight-hour advanced course includes other hemostatic methods, splinting of fractured limbs, methods to keep patients warm, and burn care.

Bystander CPR increased as laypeople training increased (Table 2); however, the number of trained laypeople peaked in 2008, followed by a decline. This decline led to modifying the guidelines by adding a 90-minute short course, which again increased participation. Currently, there are more course varieties, such as courses specializing in child first-aid and a 45-minute course focusing only on chest compression and AED use.

After the approval of layperson AED use in July 2004, public-access AED deployment and use increased rapidly nationwide (Table 2) ^(26), (29)^. Particularly, the increasing number of AEDs in densely crowded places, such as train stations, may have resulted in increased probability of bystander AED use for OHCA in Japan ^(30)^. Increased bystander defibrillation using a public-access AED on OHCA patients has contributed to improved patient outcomes ^(31), (32)^.

Research has progressed since the introduction of Utstein-style standardized data collection methods. To our knowledge, the first studies using the Utstein style in Japan were reported in 1993 ^(33), (34), (35)^. Subsequent establishment of large-scale registries (Utstein Osaka Project; Survey of Survivors after Out-of-Hospital Cardiac Arrest in the Kanto Region [SOS-KANTO]) accelerated research that provided supporting evidence to international guidelines. For example, chest compression-only CPR has been shown to be as effective as conventional CPR, with respect to long-term neurological outcomes, based on regional registries ^(36), (37)^ and the nationwide registry ^(38), (39)^.

Prognoses of OHCA patients have gradually improved over the past several decades, as a result of policy changes and subsequent efforts (Table 3). These improvements occurred through increased bystander interventions (chest compression and AED use), a strengthened pre-hospital care system (introduction of a paramedic system and increased advance life-support, particularly early defibrillation), and standardization of resuscitation procedures.

**Table 3.** OHCA Occurrence Trends, Bystander Intervention (CPR and Defibrillation), and Prognoses.

	All transported OHCA patients^*^			Bystander-witnessed cardiogenic OHCA^*^					
	Patient number	Bystander CPR provided, %	One-month survival, %	Patient number	Bystander CPR provided, %	One-month survival, %	Survival with OPC/CPC 1-2, %	Those receiving bystander defibrillation with public-access AED	
Year								Patient number	Survival with OPC/CPC 1-2, %
1994	31,206	13.4	2.6						
1995	72,016	13.0	2.7						
1996	72,542	15.1	2.7						
1997	76,272	16.9	2.8						
1998	80,970	19.7	3.2						
1999	83,353	23.0	3.2						
2000	84,899	24.9	3.4						
2001	88,058	26.6	3.3						
2002	91,691	27.8	3.5						
2003	94,845	30.8	3.7						
2004	94,920	33.5	3.9						
2005	102,738	33.6	4.3	17,882	41.0	7.2	3.3	46	23.9
2006	105,942	35.3	4.7	18,897	42.9	8.4	4.1	144	29.2
2007	109,461	39.2	5.2	19,707	47.6	10.2	6.1	287	35.5
2008	113,827	40.7	5.3	20,769	48.0	10.4	6.2	429	38.2
2009	115,250	42.7	5.6	21,112	51.3	11.4	7.1	583	35.8
2010	123,095	42.7	5.9	22,463	49.8	11.4	6.9	667	38.2
2011	127,109	43.0	5.6	23,296	49.5	11.4	7.2	738	38.9
2012	127,866	44.3	5.8	23,797	51.5	11.5	7.2	881	36.0
2013	123,987	44.9	6.1	25,469	51.1	11.9	7.9	907	42.8
2014	125,951	47.2	6.1	25,255	54.2	12.2	7.8	1,030	43.3
2015	123,421	48.1	6.3	24,496	55.8	13.0	8.6	1,103	46.1
2016	123,554	48.9	6.7	25,569	56.1	13.3	8.7	1,204	45.4	
2017	127,018	49.9	6.6	25,538	56.6	13.5	8.7	1,260	45.7
2018	127,718	50.7	6.8	25,756	58.1	13.9	9.1	1,254	48.2

AED, automated external defibrillator; CPC, cerebral performance category; CPR, cardiopulmonary resuscitation; OHCA, out-of-hospital cardiac arrest; OPC, overall performance category * Data source: Fire and Disaster Management Agency. [Report on emergency medical service and rescue, 2018]. Tokyo: the Agency; 2019. Japanese ^(26)^; the national database has registered all OHCA patients since July 1994; used the Utstein style since January 2005

## 5. Unresolved Questions

Despite these achievements, there are still challenges to be addressed in the future (Table 4). First, about half of OHCA patients still do not receive bystander CPR. In addition to enhancing CPR training for the public, barriers to performing CPR should be reduced ^(40)^. A Good Samaritan law, which currently does not exist in Japan, would dismiss concerns that one may be penalized for failing to save a life. Although, in practice, rescuers acting in good faith will not be penalized ^(41)^, such concerns may discourage people from providing CPR. Furthermore, psychological counseling for rescuers is a pressing need, as they often suffer post-CPR stress reactions ^(42), (43)^.

**Table 4.** Future Challenges.

Increase bystander CPR (still around 50% of patients receive bystander CPR). This would require:
・Training laypeople in first aid, including CPR
・Rescuer-protection mechanisms (legal, social, and psychological)
Increase the use of public-access AED by layperson bystanders (slow increase in use of public-access AED compared to rapid increase of public-access AED deployment)
Equalize pre-hospital and hospital care abilities (regional variations exist)
Facilitate and evaluate post-resuscitation intensive care (hypothermia and extracorporeal cardiopulmonary resuscitation)
Provide resuscitation according to patients’ wishes (poor management of end-of-life care)

AED, automated external defibrillator; CPR, cardiopulmonary resuscitation

Second, public-access AED use is still relatively rare, compared to the considerable number of deployed AEDs in public places ^(26)^. Location mismatch may exist, as OHCAs may be occurring in places with no or poor access to AEDs ^(44)^. Therefore, we need to identify potential places where OHCAs tend to occur, and determine the actual use of currently deployed public-access AEDs, to minimize the possibility of location mismatch. The current national registry system does not record bystander public-access AED use for cases with non-shockable rhythms. Such cases should also be registered to know total AED use.

Third, there are regional variations in patients’ outcomes ^(45)^, probably reflecting different pre-hospital care, in-hospital intensive care abilities, and data collection procedures. For example, a few areas with physician-staffed ambulance systems have shown good performance ^(33)^. This system, despite its potential, is not feasible to most municipalities, due to insufficient numbers of emergency physicians, as the policy debate indicated. Furthermore, different criteria to determine whether to resuscitate and transfer patients to the hospital (such patients are included in the national registry) might have contributed to regional variations. The proportion of non-transported or non-resuscitated cases (obvious death) differed considerably by region ^(22), (35)^. Therefore, it is crucial to standardize inclusion criteria in the national registry for more accurate evaluation, in addition to equalizing care abilities, to minimize regional variations.

Fourth, vigorous evaluation studies of in-hospital intensive care, including extracorporeal cardiopulmonary resuscitation and targeted temperature management, are needed. Such intensive care might improve post-resuscitation outcomes. Currently, however, evaluations are mostly based on observational studies, given the difficulties of experimental studies such as traditional randomized controlled trials, in critical situations. Thus, alternative study designs that may help overcome current difficulties should be considered ^(46)^.

Finally, redesigned end-of-life care management is needed in Japan’s super-aging society, where most OHCA patients are aged 80 and over, such that resuscitation attempts are performed based on the patient’s wishes. Some patients receive resuscitation attempts at the end of their lives against their wishes, due to insufficient preparation of advance end-of-care directives, insufficient communication between family physicians and specialists in emergency care, and inappropriate ambulance use. In Japan, EMS crews are not allowed to withhold or withdraw resuscitation attempts. Once they receive an EMS call, they are supposed to resuscitate and transport OHCA patients to the hospital, regardless of the patient’s wishes.

## 6. Conclusions

Outcomes for OHCA patients have improved during the past several decades, reflecting policy commitment to introduce a task-shift model in pre-hospital care. Some of the authority to provide medical care that was previously strictly restricted to physicians was delegated to paramedic-level EMS personnel and even laypeople (AED use). Since some stakeholders expressed caution toward advanced procedures provided by non-physicians with limited training, this process had to progress step-by-step: law amendments, expanding training, evaluation, and re-expansion of delegated procedures. Concomitant nationwide standardized data collection greatly contributed to evaluation. Furthermore, the nationwide database expanded clinicians’ and researchers’ perspectives from hospitals to communities and facilitated research and contribution to evidence-based practices. These Japanese experiences indicate that streamlining pre-hospital care and standardized data collection are crucial to improving OHCA patients’ outcomes. However, despite progress, many issues still need to be addressed to respond to Japanese society changes, particularly the aging population and their increasing health care needs.

## Article Information

### Conflicts of Interest

None

### Sources of Funding

This work was supported by a Grant for Comprehensive Research on Life-Style Related Diseases including Cardiovascular Diseases and Diabetes Mellitus from the Ministry of Health, Labour and Welfare, Japan [Grant number: H29-Junkankitou-Ippan-009]. The funding organization played no role in the study; the views expressed in this paper do not reflect the Ministry’s views.

### Acknowledgement

We thank Prof. Seizan TANABE for his help in obtaining literature.

### Author Contributions

SN and TS conceived the idea, all authors contributed to collecting information and interpretation of the information, SN drafted the manuscript, and all authors contributed to revising the manuscript.

### Presentation

Parts of this study were presented at the annual meeting of the Japanese Society of Reanimatology in 2017 (25-26 November 2017, Tokyo) and the annual meeting of the Japanese Association for Acute Medicine in 2018 (19-21 November, Yokohama).
